# Supplementary material for: Mortality and associated risk factors in patients hospitalized due to COVID-19 in a Peruvian reference hospital
Source: PLoS One. 2022 Mar 2;17(3):e0264789. doi: 10.1371/journal.pone.0264789 (PMC8890646; doi:10.1371/journal.pone.0264789)
Supplement: Attachment — Submitted filename: Response to reviewers.docx [file pone.0264789.s002.docx]

Lima, 24^th^ January 2021

Prof. Raffaele Serra, M.D., Ph.D

Academic editor

PLOS ONE

Dear Editor:

Following the reviewers and editor evaluation of the original research article entitled “Mortality and associated risk factors in patients hospitalized due to COVID-19 in a Peruvian reference hospital” we have included the suggestions in the manuscript, which is sent as separates files with and without track changes.

Below, you will find the answers to the specific comments.

1. Please ensure that your manuscript meets PLOS ONE's style requirements, including

those for file naming.

**Answer**

We have updated the article formatting to fulfill PLOS ONE´s style requirements

2. Please provide additional details regarding participant consent. In the ethics statement in the Methods and online submission information, please ensure that you have specified (1) whether consent was informed and (2) what type you obtained (for instance, written or verbal, and if verbal, how it was documented and witnessed). If your study included minors, state whether you obtained consent from parents or guardians. If the need for consent was waived by the ethics committee, please include this information. If you are reporting a retrospective study of medical records or archived samples, please ensure that you have discussed whether all data were fully anonymized before you accessed them and/or whether the IRB or ethics committee waived the requirement for informed consent. If patients provided informed written consent to have data from their medical records used in research, please include this information.

**Answer**

The study was limited to the retrospective review of medical records. Collection of data were anonymized before entered in the database file. The study was approved by the Department of Medicine of HNHU and by the Institutional Ethics Committees of HNHU (letter 116-2021-CIEI-HNHU) and Universidad Ricardo Palma (PG-25-2020). The scanned version of both letters are included in the new submission.

3. We note that you have indicated that data from this study are available upon request. PLOS only allows data to be available upon request if there are legal or ethical restrictions on sharing data publicly. For more information on unacceptable data access restrictions, please see <http://journals.plos.org/plosone/s/data-availability#loc-unacceptable-data-access-restrictions>.

**Answer**

Due to a misunderstanding, we believe that the standard procedure was giving the data upon request. However, we are willing to submit the database of the study. Therefore we will upload the anonymized dataset.

**Reviewer Comments to the Author**

Reviewer #1: The authors aimed to determine risk factors for in-hospital mortality in patients with COVID-19 from a Peruvian national hospital.
The findings are interesting but there are no valid comments on cardiovascular disease (except hypertension) that are important factors for mortality in COVID-19 patients. I think in the discussion section the importance of cardiovascular disease must be deepened. For example cite and discuss the paper by Ielapi N. et al. Cardiovascular disease as a biomarker for an increased risk of COVID-19 infection and related poor prognosis. Biomark Med. 2020 Jun;14(9):713-716.
Overall considered discussion section is too superficial and must be better tailored on the main aspects of the paper.

**Answer**

We have updated and improved the discussion, expanding the scope including relevant studies regarding cardiovascular and metabolic diseases. We added several additional references including the one suggested by the reviewer.

While revising your submission, please upload your figure files to the Preflight Analysis and Conversion Engine (PACE) digital diagnostic tool, <https://pacev2.apexcovantage.com/>. PACE helps ensure that figures meet PLOS requirements.

**Answer**

We have updated the figures using the PACE tool. They are now compatible with PLOS requirements.
